# Vertebrate-conserved Schnurri zinc fingers restrain Drosophila vein patterning

**DOI:** 10.17912/micropub.biology.001066

**Published:** 2024-01-26

**Authors:** Katrina Toraason, Nathan Johnson, Jenna Guernsey, Allen Laughon

**Affiliations:** 1 Undergraduate Program in Genetics, University of Wisconsin–Madison, Madison, Wisconsin, United States; 2 Department of Genetics, Department of Medical Genetics, University of Wisconsin–Madison, Madison, Wisconsin, United States

## Abstract

The *Drosophila* Smad-interacting co-factor, Schnurri (Shn) confers transcriptional repression in response to Decapentaplegic (Dpp) signaling. Shn zinc fingers 6-8 mediate this Smad interaction but are lacking in vertebrate Shn homologs. In contrast, the vertebrate-conserved zinc finger 1,2 and 4,5 pairs have been reported to engage in Smad-mediated transcriptional activation in fly and vertebrate systems, and to contribute to Dpp-dependent tissue repair in the fly retina. We report that mutation of zinc coordination residues within vertebrate-conserved Shn zinc finger pairs 1,2 and 4,5 results in ectopic venation that is sensitive to Dpp signaling.

**Figure 1. Mutations that disrupt Schnurri zinc finger pairs 1,2 and 4,5 in Drosophila result in ectopic wing venation.**
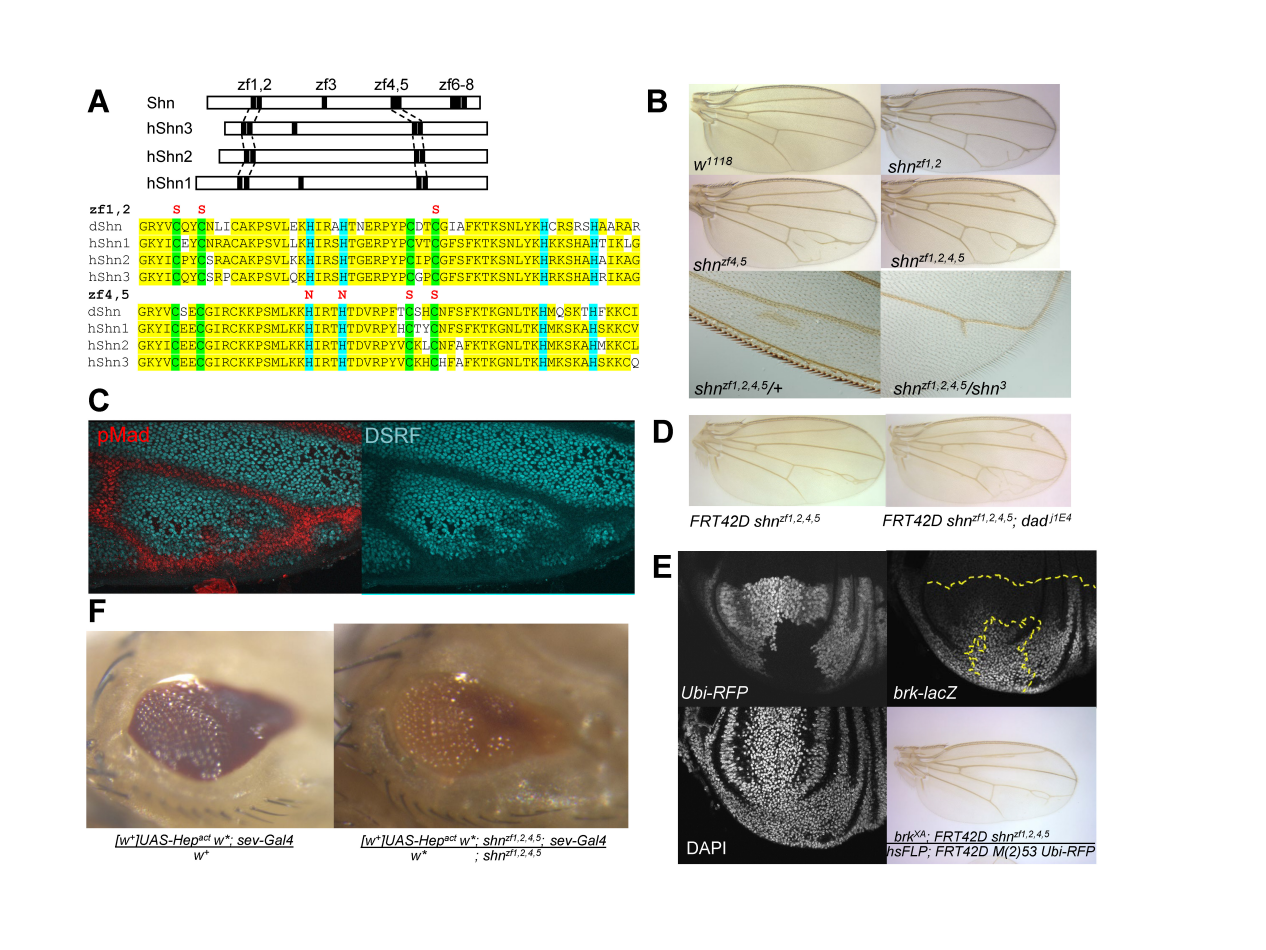
**(A)** Homologous zinc finger pairs in Drosophila Shn and human Shn family members. Shown below are the cys>ser and his>asn amino acid substitutions introduced into Shn zinc finger pairs 1,2 and 4,5 together with alignment illustrating the extensive homology with corresponding human Shn1, Shn2 and Shn3 zinc fingers (highlighted yellow). **(B)** Wings from wildtype, *shn ^zf1,2^ , shn ^zf4,5^* and *shn ^zf1,2,4,5^* females, a short patch of ectopic vein adjacent to L2 in a *shn ^zf1,2,4,5^ /+* female, and L4 branching in a *shn ^zf1,2,4,5^ /shn ^3^* female. **(C)** *shn ^zf4,5^* pupal wing stained to detect pMad (red; marks pre-vein) and DSRF (cyan; marks intervein tissue). The observed reciprocal patterns of ectopic pMad and reduced DSRF territory in pupal wings resemble the patterns of ectopic venation observed in adult wings. **(D)** Comparison of ectopic venation in *FRT42D shn ^zf1,2,4,5^* and *FRT42D* *shn ^zf1,2,4,5^ ; dad ^j1E4^* (note that severity of the *shn ^zf1,2,4,5^* phenotype was somewhat reduced in a *FRT42* background) *.* **(E)** Third instar wing disc expression of *brk ^XA^* , marked by β-galactosidase, is not reduced in *shn ^zf1,2,4,5^* clones. Large *FRT42D M(2)53 ^+^ shn ^zf1,2,4,5^* clones are marked by absence of *Ubi-RFP* (clone boundaries marked by yellow dashed lines in *brk-lacZ* panel). Clones were induced in *y ^1^ w brk ^XA^ /FM7 _i_ ; P{w ^+^ act-GFP}* ; *FRT42D shn ^zf1,2,4,5^ / y w hsFLP; FRT42D P{w ^+^ piM}45F M(2)53 P{Ubi-RFP, w ^+^ }60E* larvae *.* A resulting adult wing exhibits clonal ectopic venation. **(F)** *shn ^zf1,2,4,5^* fails to increase ommatidial loss caused by sevenless-Gal4 driven expression of an activated form of Hemipterous. Left panel: P{w+UAS-Hep.Act} ^1^ , w*/+; P{ry+GAL4-Hsp70.sev} ^332.5^ , *M{UAS-nlsTimer-NA} ^ZH-86Fb^ /+* . Right panel: P{w+UAS-Hep.Act} ^1^ , w*/ w*; shn ^zf1,2,4,5^ ; P{ry+GAL4-Hsp70.sev} ^332.5^ , *M{UAS-nlsTimer-NA} ^ZH-86Fb^ /+* . Eye color difference is due to a [ *w+* ] element that fails to fully rescue *w-* . Both crosses were done at 19 ^o^ C and were semi-lethal with a fraction of the *sev-Gal4 >UAS-Hep ^act^* progeny dying as pupae, some partially eclosed.

## Description

In response to Dpp (a *Drosophila* member of the BMP-TGFβ family), Smad proteins Mothers Against Dpp (Mad) and Medea (Med) form a complex that activates some target genes while repressing others (Hamaratoglu et al., 2014) . Repression occurs by binding of Mad-Med trimers to silencer elements (SE) that allows recruitment of Shn, a large zinc finger protein that interacts with additional co-repressors (Müller et al., 2003; Pyrowolakis et al., 2004; Gao et al., 2005; Cai and Laughon, 2009). Shn binds the Mad-Med-SE complex via zinc fingers 6-8 without apparent involvement of zinc fingers 1-5 ( Fig. 1A ). Vertebrate Schnurri proteins ( *e.g.* , human Shn1, Shn2 and Shn3) contain zinc finger pairs homologous to Shn zf 1,2 and zf 4,5 but lack counterparts to the more C-terminal zf 6-8 (Arora et al., 1995; Grieder et al., 1995; Staehling-Hampton et al., 1995; Blitz and Cho, 2009; Fig. 1A ). Unlike Shn zinc fingers 6-8, zinc finger pairs 1,2 and 4,5 have intrinsic DNA-binding specificity that resembles that of Rel proteins (Fan and Maniatis, 1990; Baldwin et al. 1990, van’t Veer et al., 1992; Dai et al., 2000). Fly Shn was found to synergize with Mad and Med in activating expression via such κB-like sites in a *Ubx B-lux* reporter (Dai et al., 2000) , evidence of functionally direct Shn-DNA contact. Experiments in Xenopus and mammalian systems found Shn proteins contributing to BMP-induced transcriptional activation by interaction with DNA-bound Smad complexes (Jin et al., 2006; Yao et al., 2006; Javier et al., 2012) . Shn has also been identified as contributing to mitigation of JNK-induced tissue damage to the fly retina, a property localizing to the region of Shn spanning zinc fingers 3-5 (Kelsey et al., 2012) . This repair/recovery of photoreceptor cells was found to be dependent on Dpp signaling through Mad (Kramer et al., 2021) .

To investigate the function of Shn zinc finger pairs 1,2 and 4,5, CRISPR-Cas9 gene editing was used to disrupt zinc coordination ( Fig. 1A ). For a variety of zinc finger proteins, it has been found that even a single Cys to Ser mutation disrupts zinc coordination and function, including DNA binding (Redemann et al., 1988; Severne et al., 1988; Witte et al., 1988; Webster et al. 1991 ). For zinc fingers 1 and 2, serine substitutions were introduced in place of three zinc-coordinating cysteines (two in zf1, one in zf2). Separately, asparagine substitutions were introduced in place of the two zinc-coordinating histidines in zf4, along with serine substitutions for the two zinc-coordinating cysteines in zf5. A resulting zf4,5 mutant line was then targeted to add the three zf1,2 substitutions, resulting in lines with a total of 7 substitutions predicted to disrupt zinc coordination by both zinc finger pairs. For all constructs, multiple correctly targeted fly lines were confirmed by DNA sequencing.

The resulting *shn ^zf1,^* ^2^ , *shn ^zf4,5^* and *shn ^zf1,^* ^2,4,5^ lines were homozygous viable with completely penetrant ectopic venation extending from or neighboring L2 and L4 ( Fig. 1B ) and phenotypic severity of s *hn ^zf1,^* ^2,4,5^ > *shn ^zf4,5^* > *shn ^zf1,^* ^2^ . With low penetrance, heterozygotes displayed small ectopic patches of vein tissue limited to the intervein territories adjacent to L2 and L4, while complementation tests against the amorphic allele, *shn ^3^* (aka *shn ^TD5^* ), resulted in mild ectopic venation with incomplete penetrance. Together, these results suggest a dosage-dependent gain-of-function in Shn activity that is competed by wild-type in heterozygotes. The mutations have no obvious effect on viability (e.g., *shn ^zf1,2,4,5^* homozgyotes recovered as a third or more of progeny from *shn ^zf1,2,4,5^ /CyO* parents) with no discerned effect on developmental timing, life span or fertility.

The characterized role of Dpp signaling in L2 and L4 formation and positioning (de Celis, 1997; reviewed by Blair 2007) , suggests that the zf1,2 and zf4,5 mutations result in an elevated response to Dpp signaling in the context of vein pattern. Consistent with this, anti-pMad staining of *shn ^zf4,5^* pupal discs revealed expanded pre-vein invasion at the expense of dSRF-positive intervein territory ( Fig. 1C ). If pMad acts directly to repress *dSRF* in the context of pupal vein patterning, presumably as a pMad-Med-Shn complex, the observed ectopic venation would indicate elevated transcriptional silencing resulting from disruption of Shn zinc finger pairs 1,2 and 4,5. This inference suggests that zf1,2 and 4,5 normally restrain or compete with the ability of Shn to engage in pMad-Med-Shn transcriptional silencing.

In comparison to the embryonic lethal phenotypes of classic *shn* alleles, the homozygous viable ectopic venation phenotypes suggest a function for zinc finger pairs 1,2 and 4,5 both distinct and separable from the carboxy-terminal zinc fingers 6-8 that have been found to be essential and sufficient for Dpp-dependent gene silencing in patterning during embryonic and larval development (Muller et al., 2003; Pyrowolakis et al., 2004). Ectopic *shn ^zf1,2,4,5^* venation was enhanced by a hypomorphic *dad* allele ( Fig. 1D ) that increases the activity of Mad-Med complexes in response to Dpp signaling (Tsuneizumi et al., 1997) , a result consistent with Dpp signaling activity contributing to this apparent gain-of-function *shn ^zf1,^* ^2,4,5^ phenotype.

The normal viability and fertility of *shn ^zf1,2,4,5^* flies suggests that these mutations have little or no effect on the ability of Shn to repress critical targets in other developmental contexts (Marty et al., 2000; Chen D, McKearin D. 2003; Pyrowolakis et al., 2004; Vuilleumier et al., 2010; Crocker and Erives, 2013; Beira et al., 2014) . Consistent with this, *FRT42 shn ^zf1,2,4,5^* clones in 3 ^rd^ instar wing imaginal discs showed no evident decrease in expression of *brk-lacZ* ( Fig. 1E ). Localized clonal ectopic venation was observed in resulting adult flies, consistent with pupal stage vein patterning being sensitive to disruption of zinc finger pairs 1,2 and 4,5.

The results raise questions regarding the role of the highly conserved Shn zinc finger pairs 1,2 and 4,5 in Dpp signaling and possibly other interactions yet to be discovered. First, how do zinc finger pairs 1,2 and 4,5 serve to prevent ectopic vein formation? Do they dampen or compete with the ability of Shn zinc fingers 6-8 to interact with silencer-bound Mad-Med complexes, perhaps by alternative Smad interactions and/or sequestering of Shn at other genomic locations? Perhaps relevant to this question, the zinc finger pairs 1,2 and 4,5 each have DNA binding affinities that overlap those of Rel proteins ( *e.g.* , NFkB) and have been found to contribute to transcription activation in vertebrate contexts. Second, the high phylogenetic conservation in vertebrates lacking homology to zinc fingers 6-8 suggests that Drosophila zinc finger pairs 1,2 and 4,5 may also function in ways that are distinct from zf6-8 mediated silencing, or one that impacts Dpp signaling conditionally, perhaps in response to stress (Clark et al. 2011) . The identification of *shn* as a loss-of-function enhancer of JNK-induced photoreceptor loss suppressible by a segment of Shn spanning zinc fingers 3-5 is suggestive of such a scenario (Kelsey et al. 2012) , although we failed to find an obvious effect of *shn ^zf1,2,4,5^* on the severity of such JNK-induced photoreceptor loss ( Fig. 1F ). Such a noncanonical role may align with studies of vertebrate Shn paralogs that mediate or intersect with BMP-regulated gene expression. Further investigation of how Shn zinc finger pairs 1,2 and 4,5 delimit vein patterning may provide mechanistic clues applicable to the diverse roles of mammalian Shn paralogs (Jones, et al. 2010; Staton et al. 2012; Takao et al. 2013; Choi et al. 2015; Srivastava et al. 2016) .

## Methods

CRISPR guide RNA target sites were chosen using the online tool CRISPR Optimal Target Finder, targetfinder.flycrispr.neuro.brown.edu (Gratz et al., 2014) . Guide RNAs for targeting zf1,2 and zf4,5 were engineered in pU6-BbsI-chiRNA by inverse PCR with Phusion polymerase (NEB) using oligos listed below. Homology repair template plasmids containing the desired point mutations were designed as previously described (Gratz et al. 2015a, 2015b; https://flycrispr.org/scarless-gene-editing/ ). First, targeted *shn* segments spanning the zf1,2 and zf4,5 regions (~2 kb for each zf pair) were PCR amplified from genomic DNA of the to-be-injected *w* ^-^ ; *vas-Cas9* stock. Next, each region was divided into “left arm” and “right arm” segments that overlap a TTAA cleavage site for piggyBac transposase that allows precise excision of an interrupting *dsRed* marker after correct targeting (see Gibson assembly primers listed below). Point mutations in zf1, zf4 and zf5 were then introduced using NEB Q5 Base-Changer site-directed mutagenesis (primers listed below). Each “arm” was then transferred into pScarlessHD-DsRed-w ^+^ by Gibson assembly (NEB Builder HiFi Assembly kit with Phusion DNA polymerase) positioning the left and right arms on opposite sides of *dsRed* (primers listed below) *.* The single zf2 cys>ser change was introduced by a primer during Gibson assembly.

For the initial set of zf1,2 and zf4,5 mutations, individual adults recovered from injected *w* ^-^ ; *vas-Cas9* embryos were crossed to *w ^-^* ; *Sco/CyO* , screening for *w ^-^* ; *dsRed ^+^* progeny indicative of successful *shn ^zf1,2/zf4,5^* targeting. Correct, error-free targeting was verified by genomic DNA sequencing extending across *shn-dsRed* junctions. Piggybac transposase was then used to precisely excise *dsRed* by crossing the balanced lines to *w* ^-^ ; *Gla/CyO; pBacT* . Resulting *dsRed ^-^* lines were sequenced across 2 kb spanning the targeted sites to verify precise *dsRed* excision and lack of spurious alterations in sequence. This yielded five correctly excised zf1,2 lines from eighteen *dsRed ^+^* lines, and three correctly excised zf4,5 lines from ten *dsRed ^+^* lines. Allelism was verified by crosses among the resulting lines, scoring for the ectopic vein phenotype. A single *w* ^-^ ; *shn ^zf^* ^4,5^ ; *vas-Cas9* line was then used to generate seven *w ^-^ ;* *shn ^zf1,2,4,5^* *dsRed ^+^ /CyO* lines (injections for this set by Best Gene). From these, seven *w ^-^ ;* *shn ^zf1,2,4,5^* lines were generated by *dsRed* excision, with sequencing verification and allelism testing.

For mosaic analysis, the *brk ^XA^* lacZ reporter was used to detect *brk* expression in *FRT42D M(2)53* ^+^ *shn ^1,2,4,5^ /* *FRT42D M(2)53* ^+^ *shn ^1,2,4,5^* clones marked by loss of *Ubi-RFP* . Immunostaining and confocal imagining of imaginal discs was performed as previously described (Blair 2000; Schleede and Blair, 2015) using the following antibodies: mouse anti-β-galactosidase (Developmental Studies Hybridoma Bank, University of Iowa), Alexa Fluor 488 conjugated goat anti-mouse IgG (Jackson ImmunoResearch); antibody reagents used to detect pMad and DSRF were as described previously (Schleede and Blair, 2015) . Sanger DNA sequencing reagents and processing were provided by the UW Biotechnology Center.

## Reagents

**Drosophila stocks**

*w* ^-^ ; *vas-Cas9* (O’Connor-Giles Lab)

*w* ^-^ ; *Gla/CyO; pBacT* (O’Connor-Giles Lab)

*w* ^-^ ; *shn ^zf1,2^* (this study)

*w* ^-^ ; *shn ^zf^* ^4,5^ ; *vas-Cas9* (this study)

*w* ^-^ ; *shn ^zf1,2,^* ^4,5^ (this study)

*w; FRT42D mago ^3^ shn ^3^ /CyO* (Bloomington 52289, donor: Nicholas Baker)

*y ^1^ w ^1118^* ; *PlacW-dad ^j1E4^ /TM3, Sb ^1^* (Bloomington 10305, source: Yuh Nung Jan)

*y ^1^ w ^111 8^ hsFLP* ; *FRT42D shn ^zf1,2,4,5^ ; PlacW-dad ^j1E4^ /TM3-TM6* (this study)

*y ^1^ w brk ^XA^ /FM7 _i_ ,P{w ^+^ act-GFP}* ; *Sco/CyO* (Bloomington 58792, source: Gerard Campbell)

*y ^1^ w brk ^XA^ /FM7 _i_ ,P{w ^+^ act-GFP}* ; *FRT42D shn ^zf1,2,4,5^* (this study)

*w* ; *FRT42D P{w ^+^ piM}45F M(2)53/CyO* (Bloomington 5698, donor: Gerald M. Rubin)

*y w hsFLP; FRT42D P{w ^+^ piM}45F M(2)53 P{Ubi-RFP, w ^+^ }60E/SM5a-TM6Tb* (this study)

P{w ^+^ UAS-Hep.Act} ^1^ , w*/FM6, w* (Bloomington 9305, donor: Marek Mlodzik)

*P{ry ^+^ GAL4-Hsp70.sev} ^332.5^ , M{UAS-nlsTimer-NA} ^ZH-86Fb^* (Bloomington 78057; donor: Peter Lidsky)

P{w ^+^ UAS-Hep.Act} ^1^ , w*/FM6, w*; shn ^zf1,2,4,5^ (this study)

w*; shn ^zf1,2,4,5^ ; P{ry ^+^ GAL4-Hsp70.sev} ^332.5^ , M{UAS-nlsTimer-NA} ^ZH-86Fb^ (this study)

**Primers for engineering gRNAs in pU6-BbsI-chiRNA** (bold = target sequence)

zf1,2gRNA-F **GTACTGACAAACGTATCGTC** GTTTTAGAGCTAGAAATAGCAAG

zf4,5gRNA-F G **TGTGCTTCTTGAGCATCGA** GTTTTAGAGCTAGAAATAGCAAG

**Primers for Q5 Base Changer site-directed mutagenesis** (bold = targeted mutation; underlined = silent change disrupting gRNA seed region)

zf1 cys1>ser_R TGA **G** A G ACGTATCGTCCGGACTTC

zf1 cys2>ser_F GTACT **C** TAACTTGATCTGTGCCAAG

zf4 his1>asn_R ATGT **T** CTT T TTGAGCATCGACGGCTTC

zf4 his2>asn_F TCGCACT **A** ACACGGACGTGAGGCCAT

zf5 cys1>ser_R GCTG **G** ATGTGAATGGCCTCACGTC

zf5 cys2>ser_F CATT **C** CAACTTCAGGTGAGTCATTG

**Primers for Gibson assembly of *shn* zf1,2 and zf4,5 genomic segments into pScarlessHD-DsRed-w ^+^** (upper case = *shn* sequence; bold = targeted mutation)

Primers for zf1,2 segment

Left arm set:

ScarlessDsRedLA_rev tcggccccgaagacacta

1-2LA_fwd tatagtgtcttcggggccgaGTGCCGCTGCCGACTGTT 38mer

1-2LA_rev (zf2 cys>ser) atatgattatctttctagggTTAAACGCAATGCCG **G** ACG 39mer

ScarlessDsRedLA_fwd ccctagaaagataatcatattgtg

Right arm set:

ScarlessDsRedRA_rev ccctagaaagatagtctgcg

1-2RA_fwd cgcagactatctttctagggTTAAGACGAAGAGTAATTTGTACAAAC

1-2RA_rev cgtatatggtcttcttttccGGCACGCACCAACTGTAA

ScarlessDsRedRA_fwd ggaaaagaagaccatatacg

Primers for zf4,5 segment

Left arm set:

ScarlessDsRedLA_rev tcggccccgaagacacta

4-5LA_fwd tatagtgtcttcggggccgaAACAGCAAGGAGGCACCG

4-5LA_rev atatgattatctttctagggTTAAAACTGTGGAATGGAACAAAG

ScarlessDsRedLA_fwd ccctagaaagataatcatattgtg

Right arm set:

ScarlessDsRedRA_rev ccctagaaagatagtctgcg

4-5RA_fwd cgcagactatctttctagggTTAAGACCAAGGGAAATCTG

4-5RA_rev cgtatatggtcttcttttccACGCCGCTGTGTATAATG

ScarlessDsRedRA_fwd ggaaaagaagaccatatacg
